# One size does not fit all: a qualitative content analysis of the importance of existing quality improvement capacity in the implementation of Releasing Time to Care: the Productive Ward™ in Saskatchewan, Canada

**DOI:** 10.1186/s12913-014-0642-x

**Published:** 2014-12-19

**Authors:** Jessica Hamilton, Tanya Verrall, Jill Maben, Peter Griffiths, Kyla Avis, G Ross Baker, Gary Teare

**Affiliations:** Health Quality Council, Saskatchewan, 241-111 Research Drive, Saskatoon, Saskatchewan S7N 3R2 Canada; King’s College London, 57 Waterloo Road, London, SE1 8WA UK; University of Southampton, Highfield, Southampton, SO17 1BJ UK; University of Toronto, Suite 425, 155 College St, Toronto, ON M5T 3M6 Canada

**Keywords:** Productive ward, Releasing time to care, Organizing for quality, Nursing, Qualitative methodology, Quality improvement capacity, Change mechanisms

## Abstract

**Background:**

Releasing Time to Care: The Productive Ward™ (RTC) is a method for conducting continuous quality improvement (QI). The Saskatchewan Ministry of Health mandated its implementation in Saskatchewan, Canada between 2008 and 2012. Subsequently, a research team was developed to evaluate its impact on the nursing unit environment. We sought to explore the influence of the unit’s existing QI capacity on their ability to engage with RTC as a program for continuous QI.

**Methods:**

We conducted interviews with staff from 8 nursing units and asked them to speak about their experience doing RTC. Using qualitative content analysis, and guided by the Organizing for Quality framework, we describe the existing QI capacity and impact of RTC on the unit environment.

**Results:**

The results focus on 2 units chosen to highlight extreme variation in existing QI capacity. Unit B was characterized by a strong existing environment. RTC was implemented in an environment with a motivated manager and collaborative culture. Aided by the structural support provided by the organization, the QI capacity on this unit was strengthened through RTC. Staff recognized the potential of using the RTC processes to support QI work. Staff on unit E did not have the same experience with RTC. Like unit B, they had similar structural supports provided by their organization but they did not have the same existing cultural or political environment to facilitate the implementation of RTC. They did not have internal motivation and felt they were only doing RTC because they had to. Though they had some success with RTC activities, the staff did not have the same understanding of the methods that RTC could provide for continuous QI work.

**Conclusions:**

RTC has the potential to be a strong tool for engaging units to do QI. This occurs best when RTC is implemented in a supporting environment. One size does not fit all and administrative bodies must consider the unique context of each environment prior to implementing large-scale QI projects. Use of an established framework, like Organizing for Quality, could highlight the distinctive supports needed in particular care environments to increase the likelihood of successful engagement.

**Electronic supplementary material:**

The online version of this article (doi:10.1186/s12913-014-0642-x) contains supplementary material, which is available to authorized users.

## Background

Releasing Time to Care: The Productive Ward™ was implemented in Saskatchewan, Canada in 2010. There was recognition that a consistent approach to quality improvement (QI) for nurses was needed as to avoid creating pockets of excellence that are isolated in one area and to ensure that projects are aligned and not competing for attention. This acknowledgement and the desire to further embed continuous QI into the daily work of those providing patient care were motivators for the provincial-wide implementation [1-4]. The Ministry of Health mandated that all surgical units in tertiary and secondary hospitals located in Saskatchewan implement RTC by March 31, 2013 [5].

RTC is built on the principles of the Toyota Productive System, often referred to as Lean [6-8], and aims to increase the autonomy of nursing staff over continuous improvement of their patient care work [1,9,10]. RTC was implemented in the United Kingdom in 2007 [9,10] and interest in the program has led to its implementation world-wide [11].

There is still limited understanding of the impact of RTC on continuous QI in the nursing environment. Alongside the implementation of the RTC program, a mixed-method evaluation was initiated to examine the effect of RTC on the unit environment, staff work-life quality, and patient outcomes. A further goal of the evaluation, and the focus of the study presented here, was to understand the short-term effect of RTC on the QI capacity of hospital units. How does the implementation of RTC develop a unit’s ability to do continuous improvement work and influence the long-term improvement outcomes expected from the program? Additionally the provincial-wide adoption of one QI program (RTC) in multiple environments (units in various Saskatchewan hospitals) gave us the opportunity to explore the impact of various unit contexts on RTC implementation. RTC is a tool-kit based program with standardized materials meant to be implemented in existing (and highly variable) working environments. We anticipated that the specific contexts of the units into which it was introduced would have important influence on the impact of the program.

There are many existing frameworks that encourage consideration of the contextual and initiative-specific factors required for successful QI in healthcare [12-17]. QI capacity is one such factor described as a key component of success with change initiatives. It is defined as an “understanding of and commitment to improvement to undertake ongoing, continuous QI work beyond any particular project” and requires knowledge and understanding of QI approaches, the ability to use data and feedback, and commitment of leadership and staff to dedicate time and resources to QI activities [18]. This description recognizes the importance of the educational aspects of QI and change practices, but also elements of context, including intentional planning for implementation, positive leadership, emotional investment and the physical infrastructure and technology to support QI work.

Despite the extensive research and many frameworks, there is still limited understanding of why some QI initiatives are successful and others are not; why a specific initiative works in one context but not in another [19,20] What is now required is to shift the focus from generating new frameworks meant to identify ‘what’ works, to using existing frameworks and identifying ‘how’ or ‘why’ a QI initiative works [20-22].

Bate, Mendel and Robert’s framework Organizing for Quality [19] moves beyond listing success factors to identifying how these factors unfold to bring about successful QI efforts. This framework is one of the first that applies organizational theories to disentangle ‘the how’ of improving quality works [19,23]. It highlights common organizational domains important to address in considering whether a group has the capacity to and is ready to engage with a QI initiative when planning and implementing a QI initiative. Within a specific environment, these domains may be negative or positive. Negative domains highlight challenges that need to be addressed while positive domains reflect potential facilitators of QI work that exist in the current environment. The six domains of the Organizing for Quality framework are described in Table 1.

**Table 1:** **Six domains of the organizing for quality framework** [19,20]

**Domain**	**Description**
Structural	The organizing and planning of quality efforts
Political	Relationships within the organization and dealing with the politics of change
Cultural	Building a shared understanding and commitment around the improvement process
Educational	Embedding and nurturing a continuous learning process
Emotional	Energizing, mobilizing and inspiring staff to join in with the QI work
Physical and Technical	The design and use of physical, informational and technological infrastructure that supports quality efforts.

The purpose of this study is to use the Organizing for Quality framework to explore the existing (pre-RTC) context and QI capacity of 8 hospital units implementing RTC and to consider the extent to which RTC helped those units strengthen or overcome limitations in their QI capacity. Where such effects were seen, we identify and describe the mechanisms by which RTC had those impacts. We performed full qualitative content analysis on all 8 units; however the results presented here focus on describing the QI capacity of the pre-RTC environment and the impact of RTC on 2 units.

## Methods

### Intervention – releasing time to care: the productive ward™ (RTC) in Saskatchewan

The United Kingdom National Health Services (NHS) Institute for Innovation and Improvement developed RTC in 2005 and 2006 and first implemented it in the UK in 2007 [8,9,23,24]. It is a self-directed tool kit consisting of three foundational modules and eight process modules. In 2008, a delegation from Saskatchewan visited the NHS to receive training and observe the use of RTC in UK hospitals. Based on this experience, the Saskatchewan Ministry of Health pilot tested RTC in Saskatchewan in fall 2008 and fall 2009 and subsequently mandated its implementation it as a province-wide program.

**Figure 1:**
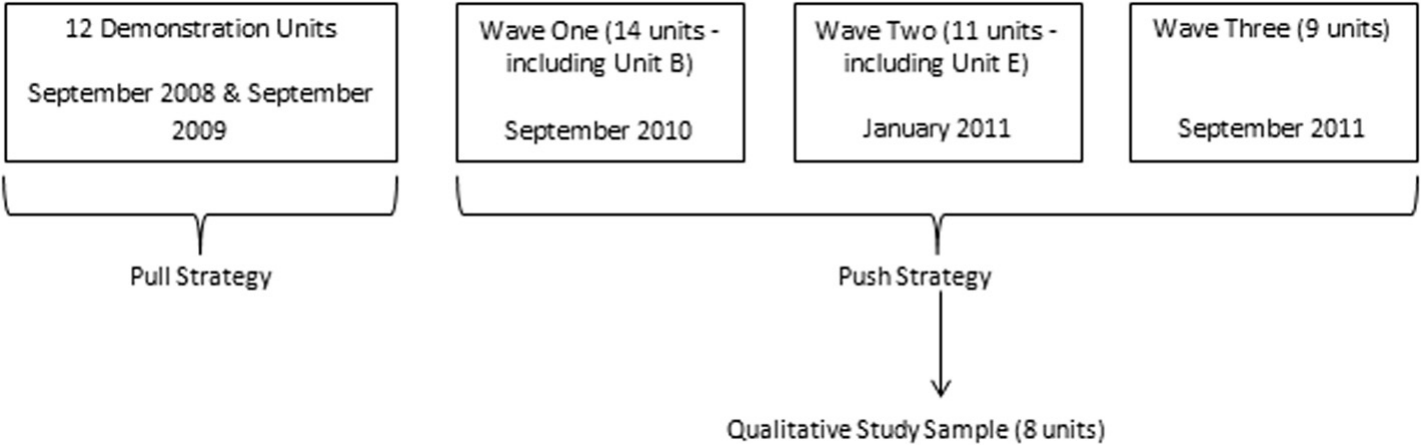
**Overview of RTC rollout in Saskatchewan.** Figure 1 provides an overview of the roll-out strategy for RTC implementation in Saskatchewan. 12 demonstration units implemented RTC starting in September 2008 and September 2009. Following this, the government mandated that all medical and surgical units in Saskatchewan hospitals implement RTC. 14 units began implementing RTC in wave 1 starting in September 2010. 11 units started implementing RTC in wave 2 in January 2011 and 9 units began in wave 3 in September 2011. These 34 units were part of the provincial RTC evaluation that was conducted alongside the government-mandated initiative. 8 of the 34 units were selected to be part of the qualitative study. Information on all 8 units is included in this manuscript but the focus is on 2 units – unit B, which started in wave 1 in September 2010 and unit E, which started in wave 2 in January 2011.

Similar to the initial roll-out in the UK, RTC implementation in Saskatchewan was originally planned as a ‘pull’ spread strategy. Between fall 2008 and fall 2009, twelve units volunteered to participate as initial demonstration units^a^. However, a ‘push’ spread strategy was adopted in April 2010, when the Ministry of Health directed the health system to implement RTC in all medical and surgical units within tertiary and secondary hospitals by March 31, 2012 [5]. Each health organization identified the medical and surgical units assigned to each implementation wave. See Figure 1 for an overview of the provincial roll-out. For purposes of the roll-out, the Ministry of Health considered units to have ‘implemented’ RTC when they had completed the three foundational modules plus one process module. Financial and QI coaching support was provided to help the units. The program was rolled out in three waves starting in September 2010. In spring 2012, support for RTC formally ended as the provincial government shifted the health system’s focus to implementation of a broader system-wide Lean-focused transformation effort led by an external consulting group [25]. At that time, sixty percent of the medical and surgical units in the province (20 out of 34) had ‘implemented’ RTC based on the definition in the provincial directive. As of fall 2014, elements of RTC still exist on some units, but most have stopped formally using the program and are focusing on the system-wide Lean transformation.

### Design and setting

This study is part of a multi-year evaluation of the implementation of RTC in Saskatchewan, Canada. The evaluation used a mixed method approach to explore the impact of RTC on the nursing unit environment and on staff and patient outcomes on the 34 units using RTC.As part of this larger evaluation, this study sought to understand the influence of existing unit context on the effect of RTC as a tool to embed continuous QI practice into the nursing environment. We utilized semi-structured interviews to answer the study’s research question.

### Sample

Interview data were collected from a purposive sample of nurses, care givers, and formal leaders associated with 8 of the 34 nursing units in Saskatchewan that were using RTC^b^ (see Figure 1). The 8 units were selected to maximize variation in geography (urban vs. rural), hospital size and duration of exposure to the RTC program. There were 4 to 7 interviews conducted on each unit, for a total of forty-eight interviews. To identify potential interviewees, we contacted individuals from each unit who were in a leadership position and familiar with the staff. The contact person was asked to identify staff from the unit who had been actively involved with RTC and staff who had been less active, including those who did not support RTC. Additionally, at the end of each interview, the researcher asked each interviewee if there was anyone else that had been a supporter or resistor of the program that we should speak with. Using this snowball sampling approach ensured we included staff with a variety of perspectives on RTC (see Table 2 for a list of interviewees from each unit).

**Table 2:** **Description of RTC units, including their pre-RTC context**

**Unit**	**Started RTC**	**RTC modules completed at time of interviews (April - June 2012) ** **(# of foundations modules** ^**a**^ **+ # of process modules)**	**Number of staff interviewed [position]**	**Pre-RTC context** ^**b**^ **(as categorized by the organizing for quality framework** **[** 19 **]** **)**
A	Wave 2 - January 2011	5 (3 + 2)	7	+/− Political
				+ Cultural
Rural, Regional Hospital		[Shift handover, Meals]	[RTC Project Lead, Unit Manager, RTC Unit Lead, Unit Clerk, Three Staff Nurses]	+ Emotional
				+ Educational
B	Wave 1 - September 2010	11 (3 + 8)	5	+ Structural
				+ Political
Urban, Provincial Hospital		[Meds; Shift Handover; Admission/ Discharge; Patient Observation; Meals; Patient Hygiene; Ward Rounds; Nursing Procedures/ Therapeutic interventions]	[Unit Manager/RTC Unit Lead, RTC co-Leads (2), Two Staff Nurses]	+ Cultural
				+ Educational
				+ Emotional
				- Physical and Technical
C	Wave 3 - September 2011	3 (3 + 0)	5	- Physical and Technical
				- Emotional
Urban, Provincial Hospital			[Unit Manager/RTC Unit Lead, Four Staff Nurses]	+ Political
				+/− Structural
				+ Educational
D	Wave 2 - January 2011	9 (3 + 6)	6	- Physical and Technical
				- Emotional
Urban, Provincial Hospital		[Meds; Shift Handover; Admission/Discharge; Meals; Patient Hygiene; Nursing Procedures]	[Unit Manager, RTC Unit Lead, Four Staff Nurses]	+/− Structural
				+/− Cultural
E	Wave 2 - January 2011	5 (3 + 2)	6	- Structural
				- Political
Urban, Provincial Hospital		[Shift Handover; Patient Hygiene]	[Unit Manager, RTC Unit Lead, Four Staff Nurses]	- Physical and Technical
				+/− Cultural
				- Emotional
F	Wave 1 - September 2010	3	4	- Physical and Technical
				- Structural
Rural, Community Hospital		(2[Well Organized Ward, Knowing How Were Doing] + 1[Shift Handover])	[RTC Unit Lead, Three Staff Nurses]	+/− Cultural
G	Wave 1 - September 2010	5 (3 + 2)	7	- Structural
				- Political
Rural, Regional Hospital		[Admission/Discharge; Shift Handover]	[RTC Project Lead, Unit Manager, Unit Coordinator, Special Care Aid, Three Staff Nurses]	- Cultural
H	Wave 1 - September 2010	7 (3 + 4)	6	- Cultural
				- Structural
Rural, Community Hospital		[Meds; Admission/Discharge; Shift Handover; Meals]	[Unit Manager/RTC Project Lead, RTC Co-Leads (2), Three Staff Nurses]	- Physical and Technical
				+/− Emotional

^a^There are three foundational modules: Well Organized Ward (WOW), Patient Status at a Glance (PSAG), and Knowing How You’re Doing (KHYD). If the unit completed all three, they are not listed. If the unit did not complete all three, the foundational modules they did complete are listed.

^b^-, + and +/− indicate that the domain was generally negative, positive, or mixed.

At the onset of each interview, the researcher explained the study to each interviewee and written informed consent was obtained. The consent letter noted that participants could withdraw at any time during or after the interview. No participants refused to participate after being approached to be part of the study, nor did any withdraw after being interviewed

### Data collection

In each interview, we asked participants to describe their involvement with RTC, their experience with implementation, and their perspectives on its success. They were also asked to describe the unit environment prior to and during RTC implementation. We used semi-structured interviews since they permitted the interviewee to respond freely and focus on issues they deemed relevant. Additionally, they allowed the interviewer the option of probing further into issues [26] (full interview guide in Additional file 1). We conducted a pilot interview with an implementation coach and the wording of the interview guide was adjusted. During this pilot, a second member of the research team observed the interview and provided feedback.

JH conducted all interviews, which ranged from 30 to 70 minutes. Interviews were recorded and transcribed verbatim. For two participants who did not want their interviews recorded the interviewer took detailed notes during these interviews. The interviewer completed 38 of the interviews in-person and 10 over the phone. All but one interview was conducted with a single participant; the exception was an interview with 2 participants. The interviewer kept a journal to document personal comments and reflections about the interviews.

### Data analysis

We analyzed the data with Atlas.ti [27] using both a directed and conventional qualitative content analysis approach [28]. In the directed approach, theming was deductive and statements were mapped to one of the domains of the Organizing for Quality framework. Though the analysis was primarily deductive, inductive coding also occurred with statements about the experience of implementing RTC that did not fit into one of the framework’s domains (conventional approach). For both the deductive and inductive coding we followed the analysis processes described by Elo and Kyngas [29]. In the preparation phase the researcher became familiar with the content of the interviews, clarified any passages that were unable to be transcribed, and paid attention to silences and tone.

We began coding and theming the interviews by unit during the organization phase. The researcher read through each passage and mapped the text to one of the domains of the Organizing for Quality framework, or used open coding if the statement did not fit one of the framework’s domains. Additionally, the researcher noted whether the description was referring to something positive (+), negative (−), or both (+/−), and to the environment prior to or during RTC implementation. During a third read through, the primary coder met with a second team member who had coded the interviews independently, following the same process. The two members read the interviews line-by-line and confirmed and clarified their coding. When the two coders disagreed, they discussed the specific quote in order to reach agreement on the themes.

We organized the themes by grouping like codes together. Themes included the six domains of the framework. The inductive analysis did not generate any unique themes. Quotes related to each major theme were then organized in separate documents. Finally, we created summary descriptions of each unit using descriptions of each theme and quotes pertaining to the unit. We completed full analysis on all 8 units; however the results presented here focus on 2 units.

### Ethical approval

This study protocol was approved by the University of Saskatchewan Behavioural Ethics Board.

## Results

Table 2 outlines the general characteristics of the 8 units, including when they started RTC, the number of modules completed at the time of the interviews, and the pre-RTC context of each unit based on the domains of the Organizing for Quality framework. Not all domains of the framework were mentioned as being relevant in the pre-RTC environment of each unit.

The results and discussion focus on 2 illustrative units, B and E. We chose these units to highlight very different examples of QI capacity in the existing (pre-RTC) context and the subsequent impact of the RTC program. Additionally, we were limited to the number of units that we could fully report on due to limitations in publication length. Though the results presented here focus on units B and E, we have included quotations relating to the pre-RTC context of units A,C, D and F-H in Additional file 2.

### Unit B

Unit B is a medical/surgical unit located in the province’s most populous health region. At the time of the study, there 46 nursing staff on the unit. This unit started RTC with the first provincial wave (September 2010). At the time of the interviews, April 2012, this unit had completed the most modules of any units (all 11 modules from the RTC toolkit). They were also completing standardized measurement associated with the Knowing How You’re Doing foundational module^c^ and were consistently submitting it to their organization for analysis.

### Pre-RTC context

When describing the unit prior to RTC, interviewees characterized unit B as having positive aspects of the **educational, structural, cultural and political domains.** However, the interviewees described the **physical and technical domain as negative** – the physical space was chaotic and unorganized.

Unit B had a number of positive components related to the **structural domain** prior to starting RTC. These included ‘education days’ where the unit manager and other staff provided training on various topics. These were offered through multiple sessions enabling all staff to attend, while patient care continued.

Positive aspects of the **educational domain** included a nurse educator who created online learning modules for staff to complete related to new initiatives or training materials. Relating to the **cultural domain**, interviewees described the atmosphere on this unit as one of existing teamwork. Staff reported that due to the nature of the work positive relationships existed between one another.

*‘We’re just used to working together lots. You just rely on your co-workers to help you get a patient up to the toilet to turn them every four hours for repositioning’. *(Staff Nurse 1)

Likewise, the staff spoke positively about the unit manager and felt they could share their ideas and concerns.

*‘She’s just one of those good leaders. Natural leader. She’s always just been very approachable’.* (Staff Nurse 1)

The unit manager described herself as being supportive of change. She was enthusiastic about starting RTC as it gave her an outlet for existing change ideas, reflecting positive factors associated with both the **cultural** and **emotional domains**.

*‘If you’re a manager that likes change, which I do, I absolutely love change, and I like process changes, and I like quality. So I think it was easy for me to step into’.* (Unit Manager/RTC Unit Lead)

Some aspects of the **physical & technical domain** were described as negative. Staff spoke of disorganization on the unit, which often resulted in them staying late to finish their duties.

*‘We were always running for stuff, we’re short this, or that lift is across the floor or the wheelchair room’s a mess. To get a wheelchair out it was a nightmare. […] We’d probably work an extra half an hour every day’.* (Unit co-Lead 1)

### Impact of RTC

The interviewees described the components of the positive existing domains, and the structural support provided by the health region, as being important for the execution of RTC. Components of the **educational, cultural and emotional domain** were strengthened. Staff reported seeing how RTC implementation helped overcome the **physical and technical challenge** as the physical space became less chaotic.

RTC, combined with the structural supports of the organization, worked within the existing environment to bring about change on the unit. The region’s QI department provided support specific to RTC and arranged for facilitators to work with this unit. The unit’s facilitator was, according to the unit manager: “*absolutely wonderful. I think that was very important to the success of our group”.* However, as time passed and the manager and staff learned from the facilitator, their own leadership ability increased and it was recognized that the facilitator was no longer as crucial to their engagement.

Additionally, the pre-existing educational days allowed the unit manager to inform staff about RTC, its goals and plan for the unit.

*‘When we did the pre-implementation we were fortunate enough that June to have education days, so the message went out to all the staff, about what Releasing Time to Care was, how it was going to be implemented, the process and what to expect out of it’.* (Unit Manager/RTC Unit Lead)

The structure of the RTC, the tool kit design, provided a focused way for the unit to trial new ideas, strengthening components of the **educational domain**. Staff described RTC as an experiential program. Implementing the modules enabled them to gain a better understanding of the program and how the process encouraged change.

*‘When you’re not really involved with RTC it is quite foreign and you don’t really know exactly what it’s about or why they’re doing it that way until you get involved with the modules and understand how it works’.* (Unit co-Lead 2)

*‘I think because they’ve all gone through the blowdryer*[improvement process model]*, and process mapping, they understand that change doesn’t just happen like that’.* (Unit Manager/RTC Unit Lead)

Through following the tool kit and RTC structure, staff noted that they were able to build on the work of their existing QI committee. The RTC process allowed staff to not only identify issues, but also delve deeper into the reasons for such problems and potential solutions. Staff came to understand the importance of using structured observations or data to drive their changes rather than relying on anecdotes and assumptions.

*‘You learn a lot, finding out from the nurses and not just guessing what is or isn’t working. You survey the staff to find out before you start making changes’.* (Staff Nurse 1)

The **educational domain** of this unit was further strengthened as interviewees reported improved staff understanding of QI and change techniques.

*‘Their minds are more open to process change […], where I think at the beginning 4 years ago, I don’t think that was there. This is now part of our culture, quality, teaching, learning, change, that’s all part of what we do now*’. (Unit Manager/RTC Unit Lead)

The **political domain** was strengthened through the staff-led focus of RTC, and the commitment of the manager to such an approach, creating a culture of empowerment and staff autonomy. The unit manager reported that doing RTC also helped her recognize the importance of supporting staff to lead the RTC work, rather than jumping to her own solutions and delegating actions. Staff noted that though they previously felt that they could share ideas with the unit manager, they appreciated her support to lead the work.

*‘The change has been directed from us, the staff. We get to pick what is not working well and we get to pick how we’re going to improve it. It’s not somebody coming on to the ward and telling us what changes need to be made when they’re not actually working every day with the patients’.* (Unit co-Lead 2)

Furthermore, some of the RTC work had a strong impact on the physical environment of the unit. The Well-Organized-Ward (WOW^d^) module helped the unit overcome the existing **physical and technical challenge**. WOW was described as critical to help staff recognize the importance of organization and efficiency, which in turn helped decrease stress and chaos. Staff reported becoming more vigilant and alert to their workspace.

*‘The Well Organized Ward module [… ]brought more calmness to the unit, because people would know where things were placed and where they were kept. It just brought more order, and when you’re well-organized, it brings less chaos to the whole environment. When you have a calmer environment you have a calmer staff, and with calmer staff, it’s easier to deal with the stresses of the unit and the stresses of the patients in the ward’.* (Unit Manager/RTC unit Lead)

The **emotional domain** was strengthened. Staff became emotionally invested in RTC and took pride in the work they had done. They became defenders of the program, encouraging engagement with the program by resistant colleagues. The morale on the unit increased as staff became more energized about their work. The changes made through RTC created calmness on the unit, resulting in staff not being as stressed out during their work hours. Staff also noted that it did ‘release time to care’ and helped improve the care that they were able to provide to patients.

*‘I am less stressed when I come to work and I do have more time for my patients. […] I’m more aware of the patients. I can remember before, thinking that there were some days I never left the desk or didn’t leave the med cart all day and now I feel like I actually get a chance to talk to my patients and find out who they are and help them solve their problems.’* (Staff Nurse 1)

Staff also described feeling like their relationships with their coworkers were strengthened, further developing a collaborative environment and strengthening the **cultural domain** of the Organizing for Quality framework.

*‘We’ve always been a tight-knit team, […] but definitely, RTC has helped us become more tight-knit’.* (Unit Co-lead 1)

In summary, this unit can be described as having strong existing (pre-RTC) QI capacity, particularly related to the **cultural, emotional and structural domains**, as evidenced by descriptions of positive teamwork and a motivational leader eager for change. Initiated in conjunction with key structural support from the health region, RTC operated within the strong environment and strengthened components of the existing environment. Teamwork was strengthened and emotional investment emerged as staff took ownership over the changes they made (**cultural and emotional domains**). Furthermore, RTC developed aspects of the **physical and technical domain**. The unit went from an existing disorganized work environment where staff felt “rushed” and “stressed” to one of “organization” and “calm”. The structure of the RTC tool kit provided a focused way for staff to make changes and through both the didactic instruction, as well as the experiential learning through trialing changes, the **educational domain** was strengthened and staff developed a better understanding of QI tools and change techniques. The RTC work helped build a foundation for continuous QI that the unit manager indicated was now part of their culture and everyday work.

### Unit E

Unit E is a medical/surgical unit located in a large health region within Saskatchewan. At the time of the study, this unit had a staff complement of 53 nurses. They began implementation of RTC in January 2011 with wave 2. At the time of the interviews in May 2012 this unit had completed 5 modules. They were no longer actively implementing RTC modules and had no plans to do any more in the future. They had also stopped collecting and submitting standardized RTC measurement data to their organization.

### Pre-RTC Context

The environment on this unit prior to RTC was characterized by **negative structural, emotional and physical and technical domains**. At the time when this unit began RTC they were also implementing a number of projects and training initiatives, contributing to an overall feeling of change fatigue. The unit’s nurse educator and the organization’s RTC project lead described them as not being ready to start RTC. The physical space was disorganized and patient care equipment was missing. The culture on the unit was described as both negative and positive.

Prior to RTC implementation staff described several initiatives that had been, or were going to be implemented. This approach suggests a lack of strategic leadership and focused plan for the various programs for the unit, highlighting negative aspects of the **structural domain**. Some staff felt this contributed to feelings of change fatigue and frustration and a lack of commitment for another initiative, or “thing to do”, highlighting an existing negative **emotional domain**.

*‘If I think back over the last few years, it’s a lot of change for units that are always on the run, I mean they’re always in crisis mode, there’s always something going on, they’re so busy. And what I’ve heard from many, all the units, it is change fatigue. There’s just too many changes, they can’t get used to the first one before you introduce the next one, and then you expect us to do another one, and another one’.* (RTC Project Lead)

A striking characteristic of this unit was the admitted lack-of-readiness by the unit’s leadership. Due to the many existing initiatives staff were overwhelmed. They were not keen to start RTC. Instead they were implementing it because they were told they had to.

*‘The general feeling was that this was mandated for the unit to do. It wasn’t something that they sought out. I think, that it would be a better way to roll-out this if actually units […] went and requested and were ready’.* [Nurse Educator/RTC Unit Lead]

Relating to the **physical and technical domain**, similar to unit B, unit E was described as being disorganized and cluttered, which resulted in staff having to spend time searching for materials. Vital patient equipment was often missing or not working. This also impacted the emotional connection staff had to their work and some staff felt they should seek out work opportunities elsewhere where basic resourcing needs were met.

*‘We have beds that don’t work, we have vital sign machines that don’t work and I think people just get fed up with it and they’re like “I want to go someplace where I have the opportunity to provide the best care possible with the best items possible with the best working machines”’.* (Staff Nurse 1)

There were mixed feelings as to the existing collaborative **culture** on the unit. Though some staff spoke of positive teamwork it was also noted that there was an environment-of-fear where staff were hesitant to share ideas or speak up.

*‘I think people would have been less inclined to speak up. I think it’s more because you don’t want to get anybody angry at you or upset with you so you just carry on with the way things go’.* [Staff Nurse 2]

Additionally, some staff felt that their leadership did not advocate for or support them, resulting in a lack-of-trust between staff and those in leadership positions. It was also observed by organizational leaders that skilled leadership on this unit was lacking.

*‘This is about leadership too, and I think that’s maybe a gap in their unit […] Why don’t they have that continuous improvement […] I think one of the biggest ones is the lack in leadership. I think they need a leader that believes that they are able to do that. They have brought lots of things to light, they’ve made suggestions, they’ve produced feedback, but it sits idle, nothing happens to it, so I don’t know that they trust’.* [RTC Project Lead]

### Impact of RTC

RTC had some impact on elements of the **cultural, emotional and physical and technical challenges** of this unit. The interviews suggest that teamwork was strengthened. Staff became temporarily emotionally invested in the program and the physical space and supporting processes were organized. Leadership continued to be an issue and the RTC program and methods were not sustained. At the time of the interviews this unit was not actively working on any of the RTC modules.

Some staff described how the teamwork approach of RTC helped to create informal leadership on the unit. Staff felt their personal confidence increased with RTC involvement.

*‘They’ve thought “Well, it’s not so dangerous to step out of our role of what we do on the floor and become a bit more responsible and lead these people to understand why we’re doing things”’.* (Staff Nurse 2)

The **cultural domain** was further developed as staff noted that they appreciated the camaraderie that developed between team members of each module as they collectively stood up for the change ideas. It was not just one person that was responsible for answering questions or taking criticism for the changes.

*‘I think the [biggest impact is the] teamwork. Showing that it could be done as a team. Showing that if you stick together, everybody stands together with what you’ve done that other people will buy-in to it more rather than just one person saying that “This is the way it should be”’.* (Staff Nurse 2)

The unit manager echoed the feeling of increased relationships suggesting that through RTC connections with the staff were strengthened. However, this feeling was not echoed by all staff. Some felt that there was no change in support from their leadership following the implementation of RTC. The unit manager also described the importance of staff ownership over the change ideas and that it was not her place to overrule the staff-made changes when they were questioned by physicians, reflecting development of positive aspects of the **political domain**.

The **emotional domain** was strengthened. Some staff described how they became emotionally invested in RTC and became defenders of the program. After investing the effort of creating and implementing changes, staff took ownership of those changes and encouraged other staff to follow suit to initiate or sustain the idea.

*‘I did say to people “Come on you guys. It’s easier if we put this here then just leaving it in the middle of the hallway or on the counter top or whatever”. […] We took ownership to it, right? So then we felt like it was our duty’.* (Staff Nurse 2)

However, this enthusiasm for RTC appeared to be temporary. This unit received QI and facilitation support from their organization for ten months. After the support period ended the unit stopped implementing the modules and there was little mention of RTC again.

*‘No, I don’t think so [whether people still talk about RTC]. I mean, once in a while there’d be mention of the Well Organized Ward Committee’.* (Staff Nurse 3)

Similar to unit B, staff reported that a successful outcome of their work with RTC was the decrease in the chaos and sense of clutter. Through the WOW Module, components of the **physical and technical domain** were strengthened. Staff were able to dedicate time and organize their equipment rooms and evaluate the amount and type of stock they kept on the unit. Staff felt that this in turn would allow them to spend more time with patients if they didn’t have to be searching for equipment or resources.

*‘I can get that dressing done quicker if I know things are in the right way so I can provide more care to more patients and more help to my fellow colleagues on different teams*’. (Staff Nurse 1)

The nurse educator reported that the process of RTC provided a focus on areas that could be improved and promoted critical thinking through a step-wise process to reflect on the issues on the unit, reflecting the **educational domain.** She felt that she would continue to use this process moving forward, even if it wasn’t formally called RTC. However, it is not evident if this idea similarly penetrated through to front-line staff. The interviews suggest that the front-line staff did not have the same understanding of the process and continuous improvement techniques that RTC could provide. They appeared to view RTC in a piece-meal fashion. It was evident that the WOW module resonated with some staff as the majority of those interviewed referred to various aspects of that module when speaking about frustrations or successes, but there was very little reference to other aspects of the RTC initiative. One nurse even questioned why they couldn’t just organize the unit instead of doing the whole of RTC. The project lead echoed this sentiment in that this was a unit that didn’t get the bigger picture of what RTC could provide.

*‘They're just not understanding how it all fits together, they say “oh yeah, there was a lady here that did RTC, and we did a bunch of change”, and they WOW'ed the unit and put some tape on the floor, but really, they don’t see the bigger picture’.* (RTC Project Lead)

The unit formally ended RTC in December 2011 after the support from their QI facilitator ended. Some staff reported that they had made good progress with RTC and there were some successes. The unit’s lead believed that more success could have been realized if they had been ready to implement.

*‘I think that was the general consensus of RTC that it was very top-driven so the whole philosophy of “We have a say and we can make changes together” is such a good one, but again, then you’re being told that “yes, this is a great philosophy but you have to do it right now” and I think that that is first and foremost the reason that it wasn’t as successful as it could be’.* [Nurse Educator/Unit Lead]

In summary, the existing QI capacity of unit E was characterized by **negative emotional, structural and physical and technical domains**, and both positive and negative components of the **cultural domain.** The unit was experiencing change fatigue due to the many initiatives being implemented and were frustrated at perceived lack-of-strategy for implementing the various programs. Additionally, somewhat negative teamwork and lack-of-trust between some staff and the unit manager contributed to a poor existing culture. The project lead identified the lack-of-leadership on the unit as a key factor in the unit’s lack of commitment to RTC. The physical space was also described as cluttered and disorganized and lacking vital resources and equipment.

This unit experienced fragmented success with RTC. In the moment of doing an individual module they appear to have experienced some engagement and achievement, particularly with the WOW module. However this did not translate into wide-spread engagement or enthusiasm for RTC as a tool for cultural change and motivation for continuous improvement. Both the RTC unit lead and project lead made note of the unit’s lack-of-readiness and the apparent push from externals to implement the program as key factors to their fragmented experience.

## Discussion

The purpose of this study was to explore the impact of RTC on QI capacity on units using the program, and the extent to which existing (pre-RTC) QI environment influenced such impact. We applied the Organizing for Quality framework to highlight the various domains important to the success or failure of this QI initiative, and to illustrate potential mechanisms of RTC. Mechanisms that help explain how RTC may have strengthened a unit’s ability to do continuous QI.

Our results suggest that RTC had a positive impact on both unit B and E, as aspects of some of the framework domains were strengthened or developed. However, the effect of RTC appears to have been stronger at unit B. At the time of the interviews, staff from unit B spoke positively about RTC. They felt it provided them with an understanding of QI theory and was an approach that could have a permanent place on their unit as a process for making constructive changes. Staff had learned numerous skills that would be transferable to other QI work, in both their current and future work environments. In contrast, staff from unit E described how, though they had some success with RTC activities, it was something they felt they had to do. This unit had completed 5 of the 11 modules. They were not actively working on any others, had not been for a while, and had no plans to continue with the other modules. At the time of the interviews, RTC had effectively ended. Unlike on unit B, there was no unit-wide recognition of the potential of RTC to act as a long-standing tool for continuous QI. Recognition of RTC as a continuous QI program is considered important to the sustainability of the program into the long-term culture [8].

We posit that the positive cultural, emotional and structural aspects of the existing (pre-RTC) QI environment may have better prepared unit B to more fully engage with RTC and further develop their QI capacity, increasing their potential to achieve long-term outcomes through their use of continuous QI techniques. These results are similar to Krein et al. [20] who found that those units with a positive emotional and cultural context were more conducive for success with QI initiatives. Unit B had a strong team environment and strong leadership, and those solid cultural aspects appear to have helped facilitate implementation of RTC. Unit E did not have the same cultural and emotional strength to support the implementation of RTC.

Our study noted the benefit of strong structural components. The QI facilitation resources for unit B allowed the unit to be able to focus on RTC and work through its processes with the help of those more experienced with QI techniques. The unit manager noted how the early-on involvement by the facilitator was crucial to their success, but as time passed, was less necessary as the unit staff developed the skills to take a more active role in leading implementation. Unit E also had a dedicated QI facilitator but they did not have the same experience with RTC. The facilitator was crucial to the RTC activities on this unit, but unlike on unit B, the staff on unit E did not appear to take on leading the RTC work as the facilitator’s dedicated time ended. Their pre-RTC context was not ready for a new QI program. There was little or no ‘pull’ for it, and despite the structural support by the organization and the potential of the program, the ability of RTC to strengthen QI capacity was weakened when placed in an environment that could not support it.

Krein et al. [20] also noted that environments that had strong emotional and cultural domains were more likely to be successful with internal motivation. Building on the work of their existing QI committee, and the energy for change that their unit manager brought, the experience described from unit B suggests that this unit was internally motivated to make changes to their unit. In comparison, data from unit E suggest that this unit was not internally motivated and staff reported that they felt like they were being externally pushed to do the program. These findings are consistent with other literature which suggests impetus for change and internal motivation are key success factor in improvement initiatives [8,10,16,30-33].

Though these key factors may be recognized within the QI and program development literature, they are less so in policy development. The government mandate that accompanied the implementation allowed us to also consider the impact of a push vs. pull spread strategy on success with QI implementation. The units that started in wave one, such as unit B, likely had existing internal motivation, were eager to implement, and often volunteered to start early on. Their health organization likely knew they were eager to begin implementation and registered them to start in the earlier waves. In contrast, unit E, and other units starting in later waves, may have felt more of a ‘push’ to start the program. Unit E reported that they felt they must implement the program and the unit lead questioned the apparent contradiction between the core idea of RTC as a nurse-led front-line initiative, and the fact that they were being told they had to do it. RTC contained an eight item checklist (readiness grid) that asked units to consider areas of leadership, competing priorities, and staff relationships. Although it is meant to be considered before beginning RTC, it was not mandatory in Saskatchewan’s implementation of RTC, nor was any formal process put in place for using it. In fact, in some cases the decision to begin implementation contradicted the recommendation from the readiness grid. Unit G is an example of this (for more details on unit G see Table 2). They completed the readiness grid, and although it indicated there may be some issues to address before starting RTC, senior leaders decided to start implementation of RTC regardless.

The push strategy may also have impacted the potential momentum and energy that could be gained through a staff-led program. Front-line ownership is a key piece of the RTC program [6,8,34-36] and our results highlight how on both units elements of this were experienced. Staff discussed becoming defenders of the changes they made and encouraging those that were resistant to become involved. In unit E, the potential impact of staff autonomy may have been minimized due to feelings of being pushed to do the program, rather than an internal motivation to begin. Though the program is designed to be led by front-line staff and decisions are to be made based on what makes sense to them, as compared to senior leaders external to the day-to-day processes, this effect may be negated by top-down pressure, as suggested in unit E.

Staff autonomy is furthermore enabled by front-line leaders who are able to act as facilitators and mentors. This was seen in unit B. The unit manager was a self- and staff- reported champion of change who described the importance of allowing staff to work through the RTC process and focus on areas they thought were important. The RTC improvement process enabled staff to systematically work through the process of identifying areas of concern and developing changes to trial. The unit manager described understanding this and that the manager role should be about providing guidance and ensuring change ideas did not violate organizational policies. The unit manager of unit E also described this understanding. However, other relationship issues existed between this manager and the staff, potentially limiting mentorship capabilities and the ability to further engage or influence staff.

The RTC tool kit and accompanying leadership training material provide advice on how leaders can best work with and support their staff during implementation. The materials support leaders to coach staff through identifying problems and brainstorming and trialing solutions, rather than trying to control and solve the unit’s issues on their own. The importance of a coaching-type leadership style is also noted in the literature [37], including other RTC reviews [36,38]. Initially, leadership was not a unique domain within the Organizing for Quality framework but rather components of leadership were captured in each of the six domains. After reviewing the appropriateness of including leadership separately, the authors have added it as a unique domain to the framework [39].

Using the Organizing for Quality Framework to describe the existing (pre-RTC) context and impact of RTC we show how, on 2 illustrative units, existing QI capacity may impact the ability of a unit to engage with a QI program and have success with it. These results highlight the importance of considering existing context when planning large-scale implementation. Scale-up of QI programs may require context-specific preparatory work in some environments prior to implementing QI programs to increase the likelihood that the investment in the program will be positive. One size does not fit all, and expectations of program success must be considered and managed when the same program is placed in varying environments. System wide implementation of like QI programs is important to create coordinated change and avoid fragmented implementation of QI, however, it is important to honour the uniqueness of each environment. By not taking the time to ensure that units, including front-line leaders, are able to support an initiative there is the risk of further damaging the culture of the unit, as well as the potential waste of time and resources. Future research should consider how to reconcile the importance of pull for a program with the realities of health care systems everywhere – government and other administrative bodies cannot wait for units to become ready and volunteer to do QI work. Further research is needed on how internal motivation and pull for QI can be developed on individual clinical units within a wider context of a whole health system that is striving to achieve patient centered transformation and improvement.

Though this study focused on the implementation of a government mandated program based on Lean principles, and not on an evaluation of Lean itself, it is important to acknowledge the critiques that exist towards Lean methodology and implementation [40-42]. As Saskatchewan embarks on a provincial wide implementation of Lean a formal evaluation is ongoing and will consider previous research of Lean [43].

The Organizing for Quality framework is one of many that can be used to assess existing content and there is continued need for the use of and refinement of existing frameworks to evaluate QI initiatives. We used this framework to describe the QI capacity of the pre-RTC context and impact of RTC on 2 extreme units. Unit B was characterized by a strong existing QI environment and the RTC program had a positive impact on their QI capacity. Unit E had a generally negative existing QI environment and they did not have a similar experience with RTC. Table 2 describes the pre-RTC context of the other 6 units interviewed and analyzed. The pre-RTC context of these units is generally characterized by both positive and negative factors. Building on these findings, future research should consider the patterns of the existing environment and explore whether a similar pattern exists as seen here with units B and E: a strong existing QI environment lends itself to positive engagement and learning, while negative existing QI environment may lead to fragmented implementation, frustration and unsustainability. How do contexts characterized by a mixed existing QI capacity fare with QI programs? How do interactions between the factors of various present or absent domains influence engagement and success with a QI program? Is there a threshold effect of QI capacity in the pre-QI program context?

Finally, our results highlight the possible mechanisms through which RTC acted upon the existing context. Staff from both unit B and E noted their appreciation of the structure of RTC and the process it provided for doing focused QI work. Through engaging with the process, staff gained an understanding of QI and change theory and techniques. The interview data suggest, however, that the understanding of QI theory and process gained was greater in unit B, perhaps because of the strong QI capacity on that unit prior to implementing RTC that provided a solid foundation to build from. In both units, collaborative culture was increased through the teamwork component of the RTC work. Staff from both unit B and E commented on how they appreciated the group approach focus of the modules. This was particularly appreciated when facing criticism from other staff. No one individual was responsible for a change. Finally, the WOW module was critical in both units. When speaking about RTC, staff consistently referred to the impact that this particular module had on their unit. It helped both units overcome their physical and technical challenges and better organize and use their space. This in turn impacted the morale of the units, decreasing the chaos and creating a sense of calm and reportedly ‘releasing time to care’. These results are similar to other studies of RTC implementation where compared to the other modules, WOW is the most visible and has the greatest overall impact [33]. Studies of Lean programs have noted the success of 5S, a similar process to sort and organize equipment and related processes [44].

Our results must be considered within the limitations of this study. The intent of this study was to explore the context on the unit prior to and during implementation of RTC. Our interviews were not longitudinal. Interviewees spoke retrospectively about the environment prior to RTC and thus there is the potential for recall bias. Our method of interview recruitment may have biased our sample as we originally relied on known people from each unit to identify key participants. This was mitigated by asking each interviewee to identify additional people to interview (snowball approach). Finally the Organizing for Quality framework notes the importance of exploring the micro, meso and macro layers of an organization. In this study, we chose to focus on the micro level, with the majority of the interviews being with front-line nurses. We did speak with each unit’s project leader who was involved in RTC at the organizational level, giving us the perspective from the meso level. We did not speak with leaders representing the macro level. Nonetheless, the richness of the detail at the micro and meso level provide strong insight into the importance of existing QI capacity, the interaction between RTC and such existing environment, and how the mechanisms of RTC can build and strengthen existing domains.

## Conclusion

The results of this study highlight the importance of understanding existing context when considering QI implementation, and the limitations of mandated top down imposed QI initiatives. One size does not fit all and when planning large-scale implementation of a single QI program attention must be given to unique contexts in order to manage expectations and identify areas where additional training and resources may be needed.

As Saskatchewan embarks on a province-wide Lean transformation the results of this study lend themselves to considering how exposure to RTC and continuous QI will impact success with Lean activity implementation. A formal evaluation of Lean in Saskatchewan is planned and it will be important that this evaluation considers the impact of RTC on the Lean transformation.

### Endnotes

^a^There was variation in how units were identified for the demonstration units – some were encouraged to volunteer by senior leaders in their health region, while in other units, the nurse manager who was familiar with RTC volunteered the unit.

^b^In Saskatchewan, ward teams consist of a nurse manager, registered nurses (RNs), Licensed Practical Nurses (LPNs), and care aids that work on a nursing unit.

^c^Knowing How You’re Doing (KHYD) is one of three foundational modules from the Releasing Time to Care: Productive Ward™ toolkit. It guides nursing units through developing ward based measures to understand ward processes and outcomes and helps teams make informed decisions [45].

^d^Well Organized Ward (WOW) is one of three foundational modules from the Releasing Time to Care: The Productive Ward™ toolkit. It provides an approach for simplifying the work area, reducing waste, and ensuring that everything is in the right place at the right time [45].

## Additional files

Additional file 1:
**Key Informant Interview Guide.** List of interview questions for the semi-structured interviews with the eight nursing units interviewed for this study. Additional file 2:
**Pre-RTC context of units A, C, D and F-H.** Example quotes for determining the domains relevant to the pre-RTC context of units A, C, D and F-H.

## Abbreviations

RTC: Releasing time to care
QI: Quality improvement
WOW: Well organized ward

## References

[1] Coutts J (2010). Accelerating excellence report: releasing time to care. Healthc Q.

[2] Avis K (2009). Releasing Time to Care in Saskatchewan: Promising Signs that the Programme Engages Clinicians.

[3] Chassin M, Loeb J (2011). The ongoing quality improvement journey: next stop. High Reliability Health Aff.

[4] Massoud M, Nielsen G, Nolan K, Schall M, Sevin C (2006). A Framework for Spread: From Local Improvements to System-Wide Change. IHI Innovation Series White Paper.

[5] Government of Saskatchewan (2010). Ministry of Health Annual Report, 2009–2010.

[6] White M, Wells J, Butterworth T: **Leadership, A key element of quality improvement in healthcare. Results from a literature review of ‘lean healthcare’ and the productive ward: releasing time to care initiative.** *Int J Leadersh Public Services* 2013, **9**(3/4).

[7] Womack J, Jones D, Roos D (2007). The Machine That Changed the World: The Story of Lean Production - Toyota’s Secret Weapon in the Global Car Wars That is Now Revolutionizing World Industry.

[8] Wright S, McSherry W (2013). A systematic literature review of releasing time to care: the productive ward. J Clin Nurs.

[9] Morrow E, Robert G, Maben J, Griffiths P (2012). Implementing large-scale quality improvement - lessons from the Productive Ward Releasing time to careTM. Int J Health Care Qual Assur.

[10] Robert G, Morrow E, Maben J, Griffiths P, Callard L (2011). The adoption, local implementation and assimilation into routine nursing practice of a national quality improvement programme: the Productive Ward in England. J Clin Nurs.

[11] White M, Wells J, Butterworth T: **The Productive Ward: Releasing Time to care™**^**TM**^**- What can we learn from the literature for implementation.** *J Nurs Manag* 2013. doi10.1111/jonm.12069. 10.1111/jonm.12069 23773544

[12] Brennan S, Bosch M, Buchan H, Green S (2012). Measuring organization and individual factors thought to influence the success of quality improvement in primary care: A systematic review of instruments. Implement Sci.

[13] Damschroder L, Aron D, Keith R, Kirsh S, Alexander J, Lowery J (2009). Fostering implementation of health services research findings into practice: a consolidated framework for advancing implementation science. Implement Sci.

[14] Dixon-Woods M, McNicol S, Martin G (2012). Ten challenges in improving quality in healthcare: lessons from the Health Foundation’s programme evaluations and relevant literature. BMJ Qual Saf.

[15] Kaplan H, Provost L, Forehle CM, Margolis PA (2012). The Model for Understanding Success in Quality (MUSIQ): Building a theory of context in healthcare quality improvement. BMJ Qual Saf.

[16] Lukas CV, Holmes SK, Cohen AB, Restuccia J, Shwartz M, Carns MP (2007). Transformational change in health care systems: An organizational model. Health Care Manage Rev.

[17] Rycroft-Malone J (2004). The PARIHS Framework - A framework for guiding the implementation of evidence-based practice. J Nurs Care Qual.

[18] Taylor EF, Genevro J, Peikes D, Geonnotti K, Wang W, Myers D (2013). Building Quality Improvement Capacity in Primary Care: Supports and Resources.

[19] Bate P, Mendel P, Robert G (2008). Organizing for Quality: The Improvement Journeys of Leading Hospitals in Europe and the United States.

[20] Krein SL, Damschroder LJ, Kowalski CP, Forman J, Hofer TP, Saint S (2010). The influence of organizational context on quality improvement and patient safety efforts in infection prevention: A multi-center qualitative study. Soc Sci Med.

[21] Bate P (2014). Context is Everything. Perspectives on Context.

[22] Rycroft-Malone J, Dopson S, Degner L, Hutchinson A, Morgan D, Stewart N, Estabrooks C (2009). Study protocol for the translating research in elder care (TREC): building context through case studies in long-term care project (project two). Implement Sci.

[23] Robert G, Anderson J, Burnett S, Aase K, Andersson-Gare B, Bal R, Calltorp J, Nunes F, Weggelaar AM, Vincent C, Fulop N, QUASER team (2011). A longitudinal, multi-level comparative study of quality and safety in European hospitals: the QUASER study protocol. BMC Health Serv Res.

[24] Wilson G (2009). Implementation of releasing time to care - the productive ward. J Nurs Manag.

[25] Marchildon G: **Implementing Lean Health Reforms in Saskatchewan.** *Health Reform Observer - Observatoire des Réformes de Santé* 2013, **1**(1). http://dx.doi.org/10.13162/hro-ors.01.01.01

[26] Green J, Thorogood N (2009). Qualitative Methods for Health Research.

[27] ATLAS.ti. Version 7.0.72. [Computer software]. (2012). Berlin: Scientific Software Development.

[28] Hsieh H, Shannon S (2005). Three approaches to qualitative content analysis. Qual Health Res.

[29] Elo S, Kyngas H (2008). The qualitative content analysis process. J Adv Nurs.

[30] Armenakis A, Harris S (2002). Crafting a change message to create transformational readiness. J Organ Change Manage.

[31] Burnett S, Benn J, Pinto A, Parand A, Iskander S, Vincent C (2010). Organizational readiness: Exploring the pre-conditions for success in organization-wide patient safety improvement programmes. Qual Saf Health Care.

[32] Hays RB, Jolly BC, Caldon LJM, McCrorie P, McAvoy PA, McManus IC, Rethans JJ (2002). Is insight important? Measuring capacity to change performance. Med Educ.

[33] NHS Institute for Innovation and Improvement, National Nursing Research Unit (2010). The Productive Ward: Releasing Time to Care Learning and Impact Review.

[34] Blakemore S (2009). How Productive Wards can improve patient care. Nurs Manag.

[35] Smith J, Rudd C (2010). Implementing The Productive Ward management programme. Nurs Stand.

[36] Morrow E, Robert G, Maben J (2014). Exploring the nature and impact of leadership on the local implementation of The Productive Ward Releasing Time to Care. J Health Organ Manag.

[37] Murphy L (2005). Transformational leadership: a cascading chain reaction. J Nurs Manag.

[38] Davis J, Adams J (2012). The Releasing Time to Care - the Productive Ward programme: participants perspectives. J Nurs Manag.

[39] Quality and Safety in European Union Hospitals (QUASER) team: **QUASER The Hospital Guide - A research-based tool to reflect on and develop your quality improvement strategies.** University College London. Retrieved from http://www.bmg.eur.nl/fileadmin/ASSETS/bmg/Quaser/QUASER-GuideForHospitals

[40] Naslund D (2008). Lean, six sigma and lean sigma: fads or reall process improvement methods?. Bus Process Manage J.

[41] Burgess N, Radnor Z (2013). Evaluating Lean in Healthcare. Int J Health Care Qual Assur.

[42] Radnor Z, Holweg M, Waring J (2011). Lean in healthcare? The unfilled promise?. Soc Sci Med.

[43] Kinsman L, Rotter T, Stevenson K, Bath B, Goodridge D, Harrison L, Dobson R, Sari N, Jeffery C, Bourassa C, Westhorp G (2014). “The largest Lean transformation in the world”: the implementation and evaluation of Lean in Saskatchewan healthcare. Healthc Q.

[44] Kim CS, Spahlinger DA, Kin JM, Coffey RJ, Billi JE (2009). Implementation of lean thinking: one health systems journey. Jt Comm J Qual Patient Saf.

[45] NHS Institute for Innovation and Improvement (2008). Releasing Time to Care: The Productive Ward Tool Kit.

