# Supplementary material for: One size does not fit all: a qualitative content analysis of the importance of existing quality improvement capacity in the implementation of Releasing Time to Care: the Productive Ward™ in Saskatchewan, Canada
Source: BMC Health Serv Res. 2014 Dec 19;14:642. doi: 10.1186/s12913-014-0642-x (PMC4279911; doi:10.1186/s12913-014-0642-x)
Supplement: Additional file 1: — Key Informant Interview Guide. List of interview questions for the semi-structured interviews with the eight nursing units interviewed for this study. [file 12913_2014_642_MOESM1_ESM.pdf]

Additional File 1: Key Informant Interview Guide

1. Can you share with me your professional background?
2. How have you been involved in Releasing Time to Care?
3. How would you describe Releasing Time to Care to someone who hasn't heard of it before?
4. a) Have you been involved in any other quality improvement initiatives?

   b) [*If yes]* Having been involved in Releasing Time to Care, do you see any differences between the design of it and the design of the other initiatives you just described?
5. Thinking back to the time before RTC was initiated on your unit - can you describe the experience of working on your unit?
6. Can you describe how RTC was introduced and rolled-out on your unit.
7. From your perspective, how did the staff on your unit react to the introduction of Releasing Time to Care?
8. Can you describe the support and resources you have for implementing RTC?
9. As a co-lead of RTC, did your leadership style change throughout doing RTC?
10. Can you describe the leadership support for RTC and its activities?
11. How prepared did you feel in your role to implement RTC?
12. In your opinion, did informal staff leaders on your unit develop throughout the course of RTC implementation?

As you may be aware, RTC is designed with three foundational modules (PSAG, WOW, KHWD) typically to be done at first followed by the process modules.

13. Can you describe the experience in implementing the foundational modules? How did you feel when you had implemented them?
14. How was the learning from the foundational modules carried over to the process modules?
15. Were there specific components of the RTC (beyond the modules themselves) that most resonated with you? That helped you understand the goals of the approach? Helped you with the approach activities? E.x. SMART goals, improvement process (hairdryer), ward review meetings]
16. As RTC was being implemented, how did the daily work on the unit change for you as the unit manager? How do you think it changed for the staff no the unit? How did the staff react to this change? Did you observe any patient reaction?

17. In your opinion, what was a) positive about doing RTC? And b) frustrating about implementing RTC?
18. What have been the barriers or challenges associated with RTC for the unit?
19. What have been the successes with RTC?
20. What do you believe is the impact of RTC implementation for you? For your unit? For the patients on your unit?
21. If you had the opportunity to repeat how you implemented RTC, do you think your unit would change the way they implemented RTC? How or what would you change?
22. Having done RTC and knowing what you know now, would you still choose to do RTC with your unit?
23. What does the future of RTC look like for you on your unit? Do you believe that doing RTC and the learning that may have come from it will impact future quality improvement work?
24. Is there anything else you would like to share regarding Releasing Time to Care and your experience with its implementation on your unit?
25. We are interested in gathering a complete picture of Releasing Time to Care implementation on your unit. Can you recommend someone we could speak to that has a different perspective about RTC than you?
