# Supplementary material for: One size does not fit all: a qualitative content analysis of the importance of existing quality improvement capacity in the implementation of Releasing Time to Care: the Productive Ward™ in Saskatchewan, Canada
Source: BMC Health Serv Res. 2014 Dec 19;14:642. doi: 10.1186/s12913-014-0642-x (PMC4279911; doi:10.1186/s12913-014-0642-x)
Supplement: Additional file 2: — Pre-RTC context of units A, C, D and F-H. Example quotes for determining the domains relevant to the pre-RTC context of units A, C, D and F-H. [file 12913_2014_642_MOESM2_ESM.pdf]

Additional File 2: Pre-RTC context of units A,C,D and F-H

Example quotes for determining the domains relevant to the pre-RTC context of units A, C, D and F-H.

Units	Domain and Example Quotes		
A	**Emotional (+):**<br>*[Because] we had such a good floor. It's hard maybe for people to understand that. We've always had a really awesome-working floor.* (Staff Nurse)	**Cultural (+):**<br>*There's always a feeling here of wanting to do the best so if something's brought forward that "This is best practices somewhere, can we incorporate that here?" So there's always that sense that we're willing to change for good change.* (Unit Clerk)	**Educational (+):**<br>*I think the girls on the IHI committee* [started before RTC] *are just doing a fantastic job. New ideas are brought forward and the nurses are always given, you know, a chance for some input* (Nurse Manager)
C	**Physical and Technical (-):**<br>*Our hallways were really cluttered, you couldn't always find what you were looking for, or you'd go into our supply room where we keep our wheelchairs and never know where to look for anything. You'd stand in there for like 5 minutes, gazing around trying to find things, or even like running from one unit to another, because something wasn't at that unit, but it's at another unit, and that kind of thing, just frustrating, you know?* (Staff Nurse)	**Emotional (-):**<br>*It's frustrating to go to work every day, because you know you love what you do, it's just, it can be so much easier, I mean you feel it can be so much easier.* (Staff Nurse).	**Educational (+):**<br>*We did a lot of quality work before […] We were already collecting the data, so well on our way. We've already been doing Patient Satisfaction surveys every month so we just had to modify one question and off we were running with that.* (Unit Manager)
D	**Physical and Technical (-):**<br>*I feel that everything was very unorganized, like when I first started I couldn't find anything on any of the carts, in any of the rooms, like you spent most of your time kind of running around and looking for things, which would be taking away from patient care.* (Staff Nurse)	**Emotional (-):**<br>*I think a lot of the new staff is for it […] I've just heard a lot of negative things about it […] I think people aren't really good with change. I've just heard people commenting how it's kind of a waste of time and money.* (Staff Nurse)	**Structural (-):**<br>*I think people are overwhelmed with changes in this region, and I mean, it's all kind of up there, and it's not well understood, until you really get into it.* (Unit Manager)
F	**Physical and Technical (-)**<br>*Disorganization, I would say. A lot of the things that we had tried to say on our committee, as far as setting up the new facility were things that the Well Organized Ward, the WOW part of Releasing Time to Care addressed, and I guess I was somewhat frustrated, I would say, in that we'd already told them those things but they didn't listen to us at that level.* (Staff Nurse)	**Structural (-)**<br>*Communication was poor, as for the start of things and stuff.* (Staff Nurse)	**Culture (+):**<br>*Generally I'd say good. Good teamwork, good relationships on the whole. (Staff Nurse)*
G	**Structural (-):**<br>*No, they didn't have consistency. I mean there was a brand new manager at the time who wasn't there for very long. They had had lots of staff turnover, which they continue to have. The manager herself was kind of like, you know I'm not ready for this, I've got, you know, there's these three or four other things that we're supposed to be starting on our unit, and then you know, like just kind of, all the things on the grid in that unit readiness grid* [RTC Project Lead]	**Political (-):**<br>*We had those ward readiness grids and I sat down, cause I knew, I mean basically it was going to be either* [this unit, or another]*, and so I sat down with the facility manager and the two unit managers at the time, and, actually I went through the grids with the unit managers individually, and went okay, well it seems pretty obvious that we should be starting with the* [other unit]*, based on what the grid is telling us, but the facility manager had already decided it was going to be* [this unit].[RTC Project Lead]	**Cultural (-):**<br>*Some of them looked at it as not necessary, as an interruption to the way things flowed and again, if it's not broke, why change it? You know, everybody kind of felt that they were going along fine.* (Nurse Supervisor)
H	**Physical and Technical (-):**<br>*I think it was a bit frenzied and very, and I think, disorganized, particularly the equipment and supplies, was kind of scattered everywhere.* (Unit Lead)	**Structural (-):**<br>*I would have liked to have seen all of our staff introduced to it just at our round table and giving them an opportunity to say "Yes, we would be interested in the program". I think it was just such a limited group of people who made that decision and yet it was going back to our staff. And the program talks about you know, "This isn't coming from above, it's not something that's being pushed on you". So I think initially if we had just involved our nursing staff. If there had have been a presentation made to them as a group I think our buy-in, our support would have been just much easier than us going out there and trying to sell the program to them.* (Unit co-Lead)	**Cultural (-):**<br>*Staff always have their idea of how we could have transitioned over here on a much smoother level and just felt that we hadn't really been listened to during the course of that change.[…] I think we've sort of come up against resistance as well from our staff because it was another program. Or they're seeing change constantly through the program and we still have some staff out there that aren't able to accept change because of what it did to them during the move.* (Unit Lead)
